# Supplementary figures and images for: Complete Genomes of DNA Viruses in Fecal Samples from Small Terrestrial Mammals in Spain
Source: Viruses. 2024 Dec 5;16(12):1885. doi: 10.3390/v16121885 (PMC11680247; doi:10.3390/v16121885)

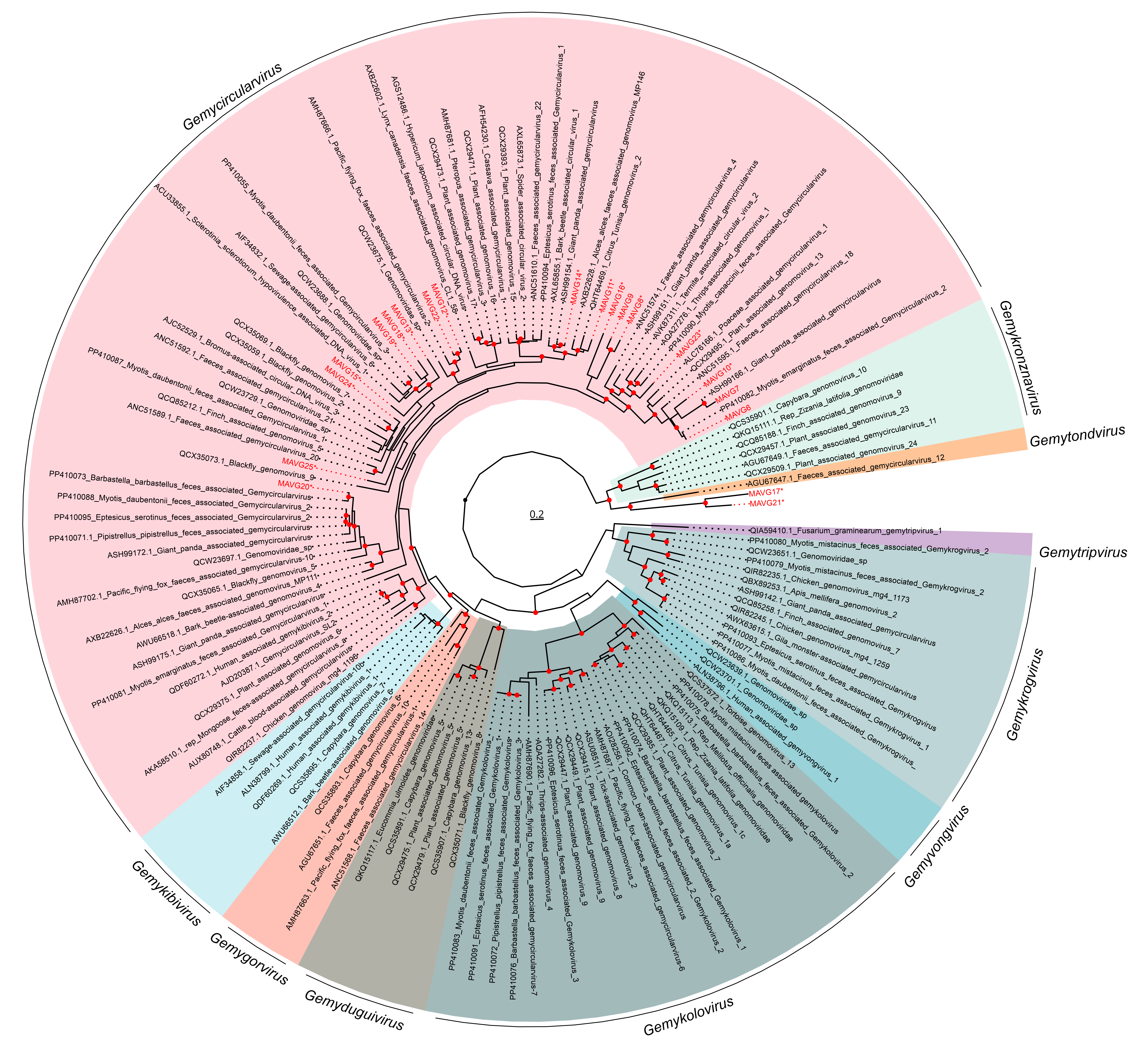

Supplement: Supplementary file 1 [file viruses-16-01885-s001.zip › Supplementary_Figure_S1.png]
